# Supplementary material for: Role of stem-like cells in chemotherapy resistance and relapse in pediatric T-cell acute lymphoblastic leukemia
Source: Nat Commun. 2025 Jun 27;16:5413. doi: 10.1038/s41467-025-61222-1 (PMC12205070; doi:10.1038/s41467-025-61222-1)
Supplement: Supplementary file 1 — Supplementary Information [file 41467_2025_61222_MOESM1_ESM.pdf]

Supplementary information

Supplementary methods

Patient-derived xenografts (PDXs)

Mouse experiments were performed under the animal licence ZH168/2021. We maintained T-ALL patient cells by intrafemoral injection of 1×10^5 to 5×10^6 viable primary ALL cells in NSG (NOD.Cg-PrkdscidIl2rgtm1Wjl/SzJ) mice. Transplanted mice were both male and female, aged 5–8 weeks. Animals were housed in individually ventilated cages with access to food and water ad libitum. Leukemia progression was monitored in the peripheral blood by flow cytometry using 1:100 anti-mouse-CD45-eFluor™ 450 (clone 30-F11, eBioscience™, Cat. No. 48-0451-82), 1:25 anti-human-CD45-Alexa Fluor® 647 (clone HI30, BioLegend, Inc., Cat. No. 304056), and 1:25 anti-hCD7-PE (clone 4H9, eBioscience™, Cat. No. 12-0078-42) antibodies. During the treatment the following items were scored: fur, habitus, body weight, activity, eyes, leukemic load and ears. Criteria for euthanization were if any item has score 3 or a total score is > 5. Especially a weight loss > 15% and a leukemic load > 75% were used as human endpoints in the treatment experiments. Mice were euthanized using CO2. The mouse was transferred into a new cage, and CO2 at4-5 l/min was introduced with a metal probe. The mouse was constantly observed and considered dead when not breathing for >1 min. The animal was observed for 2 minutes after removal from the CO2 cage, to ensure lack of breathing. T-ALL cells were collected from spleen and cryopreserved as described[1]. Xenograft identity was verified by DNA fingerprinting using the commercial AmpFlSTR® NGM Select kit.

MSC cell culture

Human hTERT immortalized primary bone marrow mesenchymal stroma cells[2] (MSC) were cultured in RPMI 1640 medium supplemented with 10% heat-inactivated fetal bovine serum, L-glutamine (2 mM), penicillin/streptomycin (100 IU/ml) and hydrocortisone (1 µM).

Drug response profiling

Drug responses were assessed in T-ALL cell co-cultures on hTERT-immortalized primary bone marrow MSCs in 384-well plates (Greiner, REF781090). 2,500 MSCs per well were plated in 20 µl AIM V medium 24 hours before adding 10,000 T-ALL cells per well in 20 µl of AIM V recovered from cryopreserved PDX samples. After 24 hours different drugs were added by using Echo 650 Series Liquid Handler (Beckman Coulter). The tested compounds included Cytarabine, Venetoclax, S-63845, A1331852 and FX1 (all from MedChemExpress), which were applied in a concentration range from 0.1 nM to 10,000 nM. After incubating the cells for 72 hours, they were stained with CyQuant and imaged using the Operetta CLS (PerkinElmer), a high-content imaging system. The captured images were processed using BIAS (Single-Cell-Technologies), an analytical software tool. Drug response parameters were determined using Non-Linear Least-Squares Minimization and Curve-Fitting for Python, a statistical method used to model dose-response relationships.

scRNA-seq: library preparation

Cryopreserved cells were thawed at 37°C and resuspended in 10 ml RPMI medium + 20 % FBS. Cells were centrifuged for 5 min at 300g and resuspended in ice-cold PBS + 2% FBS + 5mM EDTA. Cells were stained on ice and in the dark for 30 min with 1:20 anti-mouse-CD45-PE (clone 30-F11, BioLegend, Inc., Cat. No. 103106). 1:100 4,6-diamidino-2-phenylindole (DAPI) was added immediately before FACS sorting. mCD45-DAPI- cells were sorted using a BD FACSAria fusion cell sorter.

For VASA-seq, single cells were sorted into cooled 384-well plates containing primers with well-specific barcodes. After sorting, plates were immediately spun and placed on dry ice. Plates were shipped on dry ice to Single Cell Discoveries (SCD), where libraries were prepared by the company according to the VASA-seq protocol[3] and sequenced on the Nova Seq X Plus (10B - 100 cycle, paired-end, 150.000 reads/cell). VASA-seq relies on short-read sequencing to reconstruct full-length transcripts through computational assembly. Unlike single-molecule long-read sequencing approaches, such as PacBio or nanopore sequencing, which directly read long RNA or cDNA molecules, VASA-seq captures full-length transcript information by sequencing fragmented reads, offering high-throughput resolution at the single-cell level. Mapping, *in silico* depletion of rRNA and generation of count tables were automated using the STARSolo aligner (https://github.com/alexdobin/STAR/blob/master/docs/STARsolo.md). For 10x Genomics bulk of cells was sorted in ice-cold PBS + 3% BSA. Samples were processed immediately according to standard 10x Genomics Chromium 3' (v.3.1 Chemistry) protocol. Libraries were sequenced on the NovaSeq 6000 S2 v1.5 (100 cycles) flowcell S2 (Surface: 3.3-4.1 BIO reads). Transcripts were quantified into count matrices using cell ranger mkfastq and count workflows (10x Genomics, v.3.1.0, default parameters). Sequenced transcripts were aligned to a mixed human-mouse genome (GRCh38-GRCm38). The R package Seurat was used for downstream analysis[4].

scRNA-seq: Data preprocessing and quality control

Human cells were separated from mouse contaminations by filtering for cells with more than 80% of transcripts aligning to GRCh38. Remaining reads aligned to the mouse genome were removed from the analysis. Filtering of low-quality cells based on gene count (VASA-seq: >2500 and <9000, 10x Genomics: >2000 and <7500), read count (VASA-seq: <100000,

10x Genomics: <40000) and mitochondrial fraction (VASA-seq: <5%, 10x Genomics: <10%) was performed.

scRNA-seq: Dimensionality reduction and clustering based on RNA expression

Downstream processing involved log2normalization, scaling and linear dimensional reduction (PCA). We assigned cell cycle scores using the CellCycleScoring() function of *Seurat* and canonical histone expression scores using the AddModuleScore() function. Both scores were regressed out during scaling of the data. For the individual patient (see Fig. 1, Fig. 5) and the TAL1 cohort analysis (Fig. 2) graph-based clustering analysis was performed on uncorrected samples using the Louvain algorithm and visualized by UMAP. For the integrated analysis of TAL1 patients (see Supplementary Fig. 2b), samples were batch corrected using the anchor-based CCA integration of Seurat.

scRNA-seq: Gene-regulatory networks and dimensional reduction based on regulon activity

Gene-regulatory networks of VASA-seq data was inferred using an in-house constructed Snakemake pipeline[5] of the pySCENIC package[6,7]. pySCENIC is the python implementation of the SCENIC pipeline[8] and allows for a better scalability and integration with existing scRNA-seq workflows compared to the original method. The tool calculates the single cell activity of regulons in three steps: 1) generation of co-expression modules of transcription factors and genes (GENIE3). 2) removal of indirect targets using TF motif information (RcisTarget). 3) calculation of regulon activity in individual cells (AUCell). For the TAL1 cohort and the expanded cohort containing all subgroups scaling and clustering based on the Louvain algorithm were performed on the uncorrected samples using the pySCENIC regulon activity.

For differential expression of the TAL1 cohort, cells were merged into two larger clusters (Stem_like and Blasts) and the FindAllMarkers() function was applied [4] to focus on specific stem cell-like markers compared to other leukemic cells (log2FoldCange (log2FC) > 0.5 and padj < 0.05).

Generation of a Stemness Score

DE genes of the TAL1 stem-like cell population with a log2FC> 0.5 and padj < 0.05 (= 601 genes) were treated as a set of markers, which we used to calculate a stemness score for all individual T-ALL PDX-derived cells of our study (including cells from other subgroups). For this, we applied the AddModuleScore() function in Seurat. This function calculates an aggregated expression score for a predefined set of genes[4] - here, the 601 DE genes from our initial TAL1 analysis. Specifically, it computes the average expression of the gene set per cell, comparing it to a randomly selected control gene set of the same size. The resulting score reflects the relative expression of the selected DE genes within each cell. In order to identify a threshold for the definition of stem-like cells, individual cells were ranked according to their score and natural breaks in the stemness scores were identified using the getJenksBreaks (k=4) function from the BAMMtools package. The stemness score was further assessed in *in-vitro* and *in-vivo* drug-treated scRNA-seq data and differences were analyzed using a two-sided t-test. For the calculation of stemness in a bulk RNA-seq dataset of 1,336 diagnostic T-ALL samples[8], the relative expression of the 601 stemness markers was assessed using the AddGeneSetScore() function from the *geneset-modulescoring* package (https://github.com/HerpelinckT/geneset-modulescoring), which is an adaptation of the AddModuleScore() function tailored for scoring gene sets in bulk RNA-seq datasets. We

calculated a stemness score for each patient and analyzed differences based on their initial treatment response (M1: <5% blasts, M2: 5-25% blasts, M3: >25% blasts) using a two-sided t-test.

Gene Set Enrichment Analysis (GSEA)

For PDX-derived cells of the index patient P2, the FindMarkers() function was applied on the clusters that were identified by a graph-based clustering analysis using the Louvain 6lgorithm (standard Seurat workflow) to identify DE genes. For the TAL1 cohort instead, DE genes from the pySCENIC analysis have been used as input for GSEA. Only genes with a log2FC > 0.5 and padj < 0.05 (calculated using the Bonferroni correction method) were retained. To perform the GSEA, we ran the prerank() module from the gseapy package on the list of DE genes ranked by log2FC using standard parameters[9]. The module calculates an enrichment score for each gene set, assessing the degree to which members of the gene set are overrepresented at the top or bottom of the ranked list. The enrichment score is then normalized (NES) based on the gene set enrichment scores for all dataset permutations, which is the primary statistic used to compare enrichment results across gene sets. Gene sets were retrieved from the 'GO Biological Processes 2023' library.

Annotation of T-ALL PDX cells using thymic and bone marrow references

To infer which T cell progenitor stage individual T-ALL PDX cells resemble most closely, a thymic single cell atlas was used as a reference[10] and mapped onto our dataset using the functions FindTransferAnchors()and MapQuery() of the Seurat package[4]. In addition, cells were mapped onto a bone marrow single cell reference generated by the HuBMAP consortium using the RunAzimuth() function of the Azimuth package[11].

Projection of TAL1 samples to DP-like and $\alpha\beta$-like TAL1 subsets

DE genes of the DP-like and $\alpha\beta$-like TAL1 subgroups have been retrieved from the supplementary material of the Pölönen et al, Nature 2024 publication[8]. Cells of the TAL1 cohort were scored based on their relative expression of DP-like genes and $\alpha\beta$-like using the AddModuleScore() function of the Seurat package.

Alternative Splicing (AS) Analysis

First, reads were demultiplexed and trimmed with TrimGalore (https://github.com/FelixKrueger/TrimGalore). Then, the barcode-specific FASTQs were ribo-depleted using both mouse and human ribosomal-DNA sequences as reference. Reads mapping uniquely to the mouse genome (GRCm38) were eventually discarded with BBMap (https://sourceforge.net/projects/bbmap/). The quantification and differential testing of AS events were performed by applying the specialized computational workflow published in Salmen et al[3]. Firstly, we expanded the transcriptome of each PDX-derived sample. In synthesis, FASTQ pseudo-bulks, corresponding to the previously identified clusters, were aligned to the reference human genome (GRCh38) via HISAT2 with standard configuration[27]**.** The resulting transcriptomes were assembled and merged with StringTie2[12]. The newly annotated isoforms underwent several quality-control steps to filter out possible false positives (see Methods in Salmen et al[3]). Consequently, high-confidence novel isoforms were added to the reference GTF file (release GRCh38.110).The successive step consisted in further expanding the transcriptome by adding novel microexons (exons < 30 nt) to the reference GTF annotation. Therefore, we employed the discovery module from MicroExonator, a Snakemake workflow specifically designed to identify and quantify microexons[13]. The filtering of spurious hits was performed following the authors' guidelines.

To quantify AS events across cell clusters, we ran the MicroExonator's downstream module 'snakepool' with default parameters. Cells from the same cluster were randomly pooled together into 5 pseudo-bulks of equal size. The PSI (percentage-spliced-in) value returned for each splicing node in the pseudo-bulks was used to provide a probability of differential inclusion. To avoid false positives, the pseudo-bulk quantification and assessment of differential inclusion were repeated 50 times for each pairwise comparison. The probabilities of each splicing node were then fitted to a beta distribution and the CDF-beta value was returned. Splicing nodes with CDF-beta < 0.05, mean probability > 0.9, and $\Delta PSI > 0.2$ were considered differentially included, while events found significant in less than 25 repetitions were discarded. We designed the pairwise comparisons to expose the different AS patterns between the stem-like cluster and other clusters individually or collectively per patient. To quantify the splicing events at the single-cell level, we instructed MicroExonator to bypass the pseudo-bulk pooling and differential testing. Sashimi plots of specific AS events were created with the ggsashimi package[14]. Gene Ontology (GO) over-representation analysis was performed using the enrichr() module from the gseapy Python package with standard parameters. Gene sets were retrieved from the 'GO Biological Processes 2023' library.

Supplementary Figures

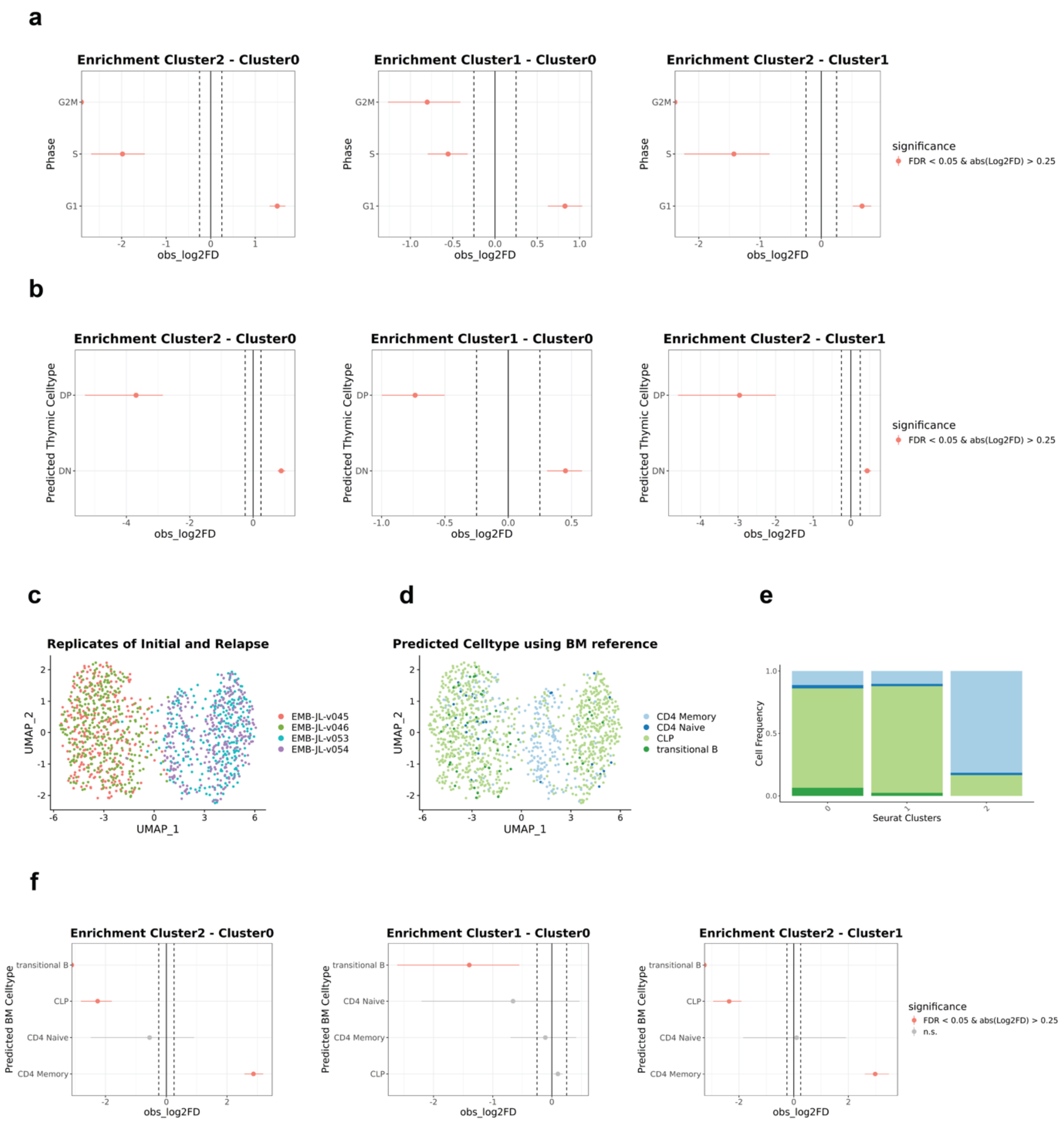

Supplementary Figure 1: **Analyses of cell cycle phases, predicted cell types and technical replicates of Patient P2.** a), b), f): Permutational analysis. N = 1000 permutations per analysis, significance threshold: FDR <0.05, log2FD > 0.25. Comparisons of enrichments from left to right) cluster 2 vs cluster 0, cluster 1 vs 0, cluster 2 vs cluster 1. a) predicted cell cycle phase enrichment. b) predicted thymic cell type enrichment. f) predicted BM cell type enrichment. c) UMAP visualization of P2 initial (EMB-JL-v045 and EMB-JL-v046) and relapse

(EMB-JL-v053 and EMBL-JL-v054) technical replicates (n = 1,231 cells from 2 biological samples). d),e): distribution of predicted cell types of the bone marrow (BM) after mapping onto a human bone marrow reference dataset[11] visualized by UMAP (n = 1,231 cells from two samples) (d) and stacked barplot (e). Source data are provided as a Source Data file.

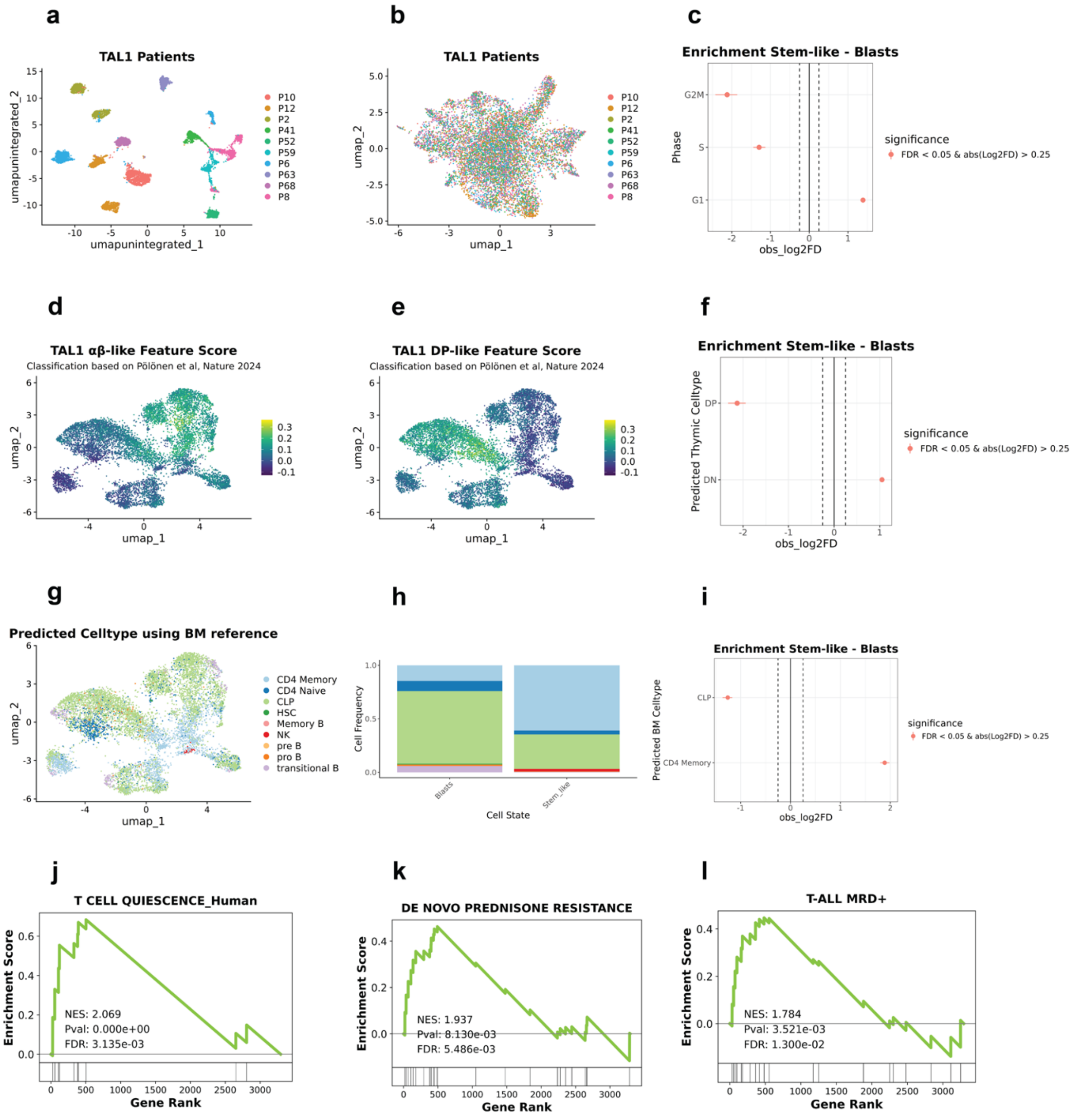

Supplementary Figure 2: **GSEA of TAL1 stem-like cells vs blasts and UMAP plots of TAL1-driven T-ALL patients, additional classification of TAL1 subgroup and predicted BM cell type**. a) Graph-based clustering analysis using the Louvain algorithm of unintegrated TAL1-driven T-ALL PDX samples using RNA expression visualized by UMAP (n = 9,160 cells from 15 samples, 10 patients). b) Graph-based clustering analysis using the Louvain algorithm of TAL1-driven T-ALL samples using RNA expression after anchor-based CCA integration

visualized by UMAP (n = 9,160 cells from 15 samples, 10 patients. c), f), i): Permutational analysis. N = 1000 permutations per analysis, significance threshold: FDR <0.05, log2FD > 0.25. Comparison of stem-like cells vs blasts. c) predicted cell cycle phase enrichment. f) predicted thymic cell type enrichment. I) predicted BM cell type enrichment. d) Scoring of relative expression of TAL1 $\alpha\beta$-like RNA features from Pölönen et al, Nature 2024 (n = 9,160 cells from 15 samples, 10 patients)[8] . e) Scoring of relative expression of TAL1 DP-like RNA features from Pölönen et al, Nature 2024 (n = 9,160 cells from 15 samples, 10 patients)[8]. g),h): distribution of predicted cell types of the bone marrow (BM) after mapping onto a human bone marrow reference dataset[11] visualized by UMAP (n = 9,160 cells from 15 samples, 10 patients (g) and stacked barplot (h). j), k), l): GSEA plots of published datasets. j) signature of T cell quiescence genes (human orthologs were used)[15].k) signature of *de novo* prednisone resistance in T-ALL[16]. l) signature of T-ALL MRD[17]. Source data are provided as a Source Data file.

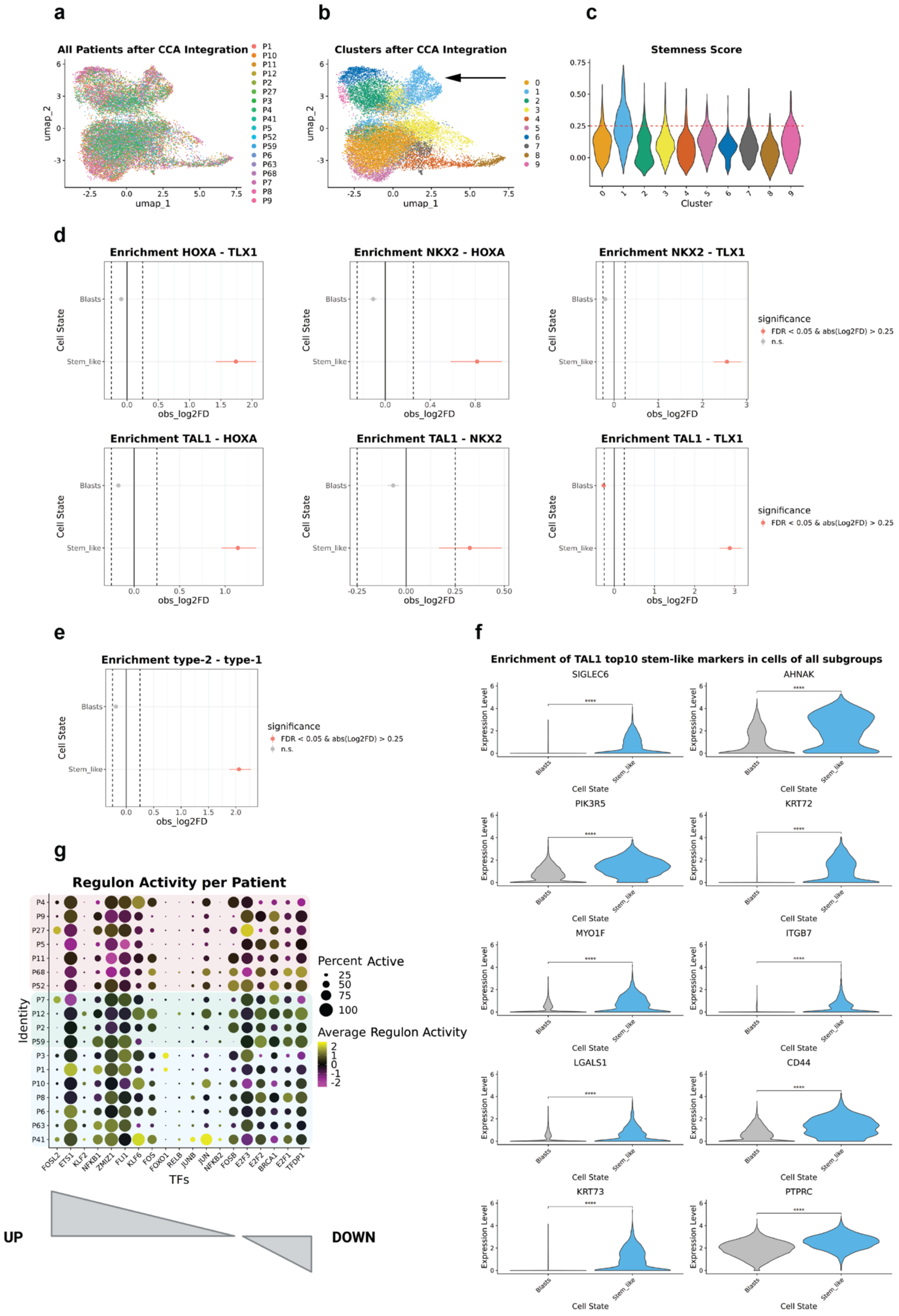

Supplementary Figure 3: **Analyses of the stem-like cell proportion among T-ALL subgroups**

and relapse types and differential expression analysis of TAL1 top10 stem-like markers. a), b): Graph-based clustering analysis using the Louvain algorithm of T-ALL PDX samples from all subgroups using RNA expression after anchor-based CCA integration visualized by UMAP (n = 18,878 cells from 31 samples, 18 patients). a) distribution of PDX-derived cells from individual patients. b) distribution of clusters. c) Stemness score displayed per cluster. Red dashed line reflects the threshold used for the definition of stem-like cells (0.25) (see Fig. 3e). d), e): N = 1000 permutations per analysis, significance threshold: FDR <0.05, log2FD > 0.25. a) enrichment comparisons of first row, from left to right: stem-like population in HOXA vs TLX1 PDX-derived samples (n = 10 samples, 5 patients), stem-like population in NKX2 vs HOXA PDX-derived samples (n = 8 samples, 4 patients), stem-like population in NKX2 vs TLX1 PDX-derived samples (n = 10 samples, 5 patients); enrichment comparisons of second row, from left to right: stem-like population in TAL1 vs HOXA PDX-derived samples (n = 21 samples, 13 patients), stem-like population in TAL1 vs TLX1 PDX-derived samples (n = 23 samples, 14 patients), stem-like population in TAL1 vs NKX2 PDX-derived samples (n = 21 samples, 13 patients). b) enrichment of stem-like population in type-2 vs type-1 relapses (n = 26 samples, 13 patients). f) 10 violin plots displaying individual expression levels (log2FC) of TAL1 top10 stem-like markers in blasts vs stem-like cells of all T-ALL subgroups. **** = $p < 2.22e^{-16}$ (two-sided t-test). g) Differential activity of TAL1 stem-like regulons in PDXs of individual patients (Supplementary Data 5) visualized by a dotplot. The size of the dot encodes the percentage of cells per patient, while the color encodes the average regulon activity level across all cells of the patient (yellow is high, purple is low). Blue, green and red background color corresponds to the different stem-like categories defined in Fig 3h). Created in BioRender. Costea, J. (2025) https://BioRender.com/z6pnqhb. Source data are provided as a Source Data file.

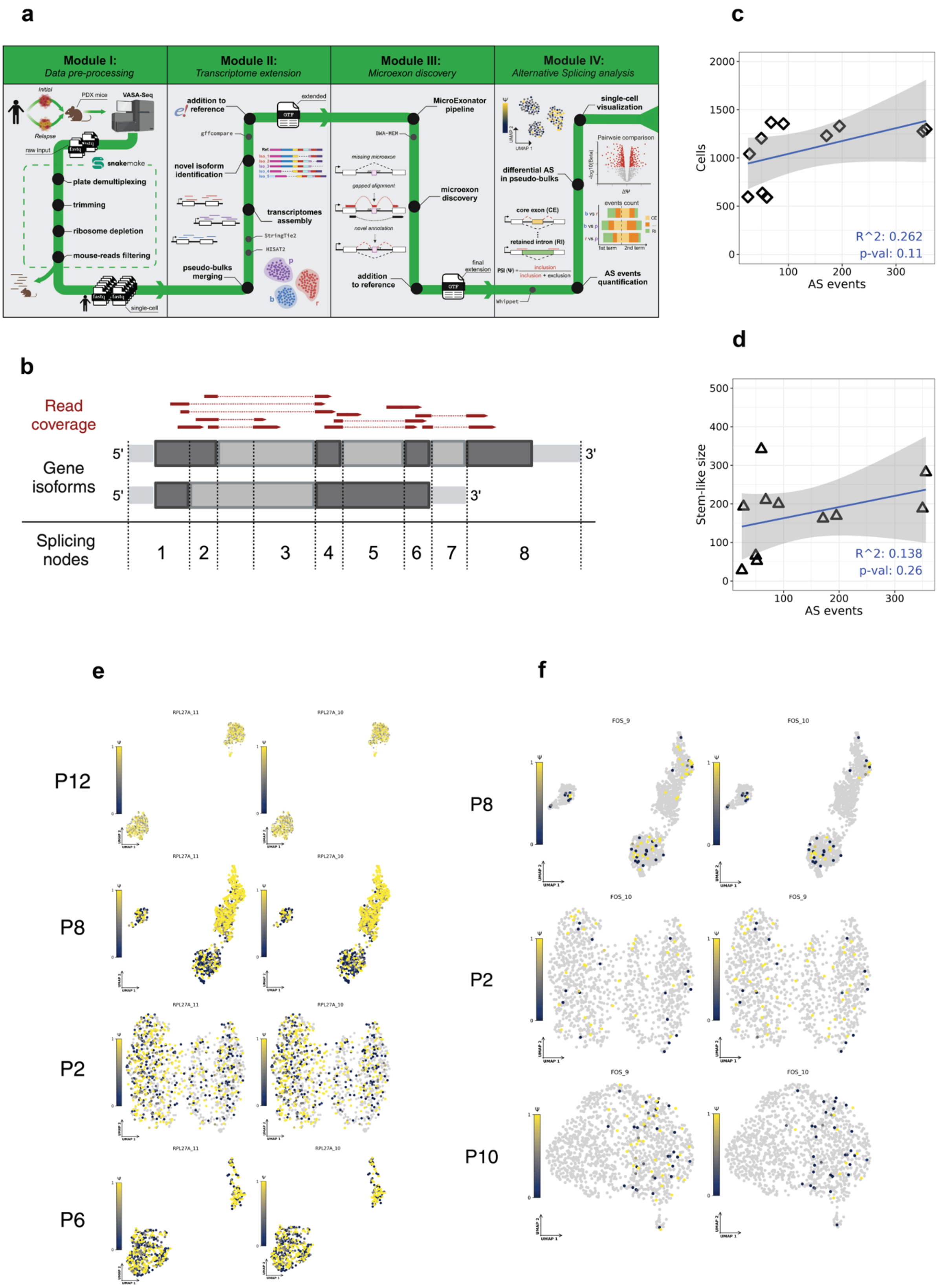

Supplementary Figure 4: **Computational workflow of AS analysis and single-cell quantification of AS events**. a) Representation of the four contiguous modules composing

the workflow for the detection of AS in the stem-like cell population. First, raw FASTQ are pre-processed and mouse-reads discarded, then the transcriptome annotation is expanded with novel isoforms and microexons inferred from the data. The last module quantifies inclusion rates (ψ) at the single-cell level and performs statistical tests to identify AS events with high confidence. Module I, II, III were adapted from Salmen et al[3]. Created in BioRender. Costea, J. (2025) https://BioRender.com/a3q0pri. b) Representation of 'splicing node', the unit of measure of the AS workflow. For each gene, UTRs, exons (dark grey), and introns (light grey) from different isoforms are collapsed into unique and non-overlapping gene intervals, denominated splicing nodes. In addition to intron/exon junctions, splicing node boundaries are also inferred from the read coverage (e.g., node 3). Created in BioRender. Costea, J. (2025) https://BioRender.com/1bolrv7. c) Correlation between AS events (x-axis) and number of cells sequenced (y-axis) for PDXs of each patient (n = 11). d) Correlation between AS events (x-axis) and size of the stem-like cluster (y-axis) for each patient (n = 11). c), d): Data was fitted through a linear model; p-value (pval) and goodness of fit (R^2) are displayed in the bottom-right corner. e), f): ψ UMAP plots of individual patients. Number of cells per UMAP: P6 (n = 1,204 from two samples), P8 (n = 1,276 from two samples), P10 (n = 1,330 from two samples), P2 (n = 1,231 from two samples), P12 (n = 1,298 from two samples). e) UMAP plots of PDX-derived cells from P12, P8, P2, and P6 on which the ψ value of RPL27A node 11 (left) and 10 (right) is projected. f) UMAP plots of PDX-derived cells from P8, P2, P10 on which the ψ value of FOS node 9 (left) and 10 (right) is projected. (e, f) Yellow is complete node inclusion (ψ=1), while blue is complete node exclusion (ψ=0). Gray cells lack information about the node. Source data are provided as a Source Data file.

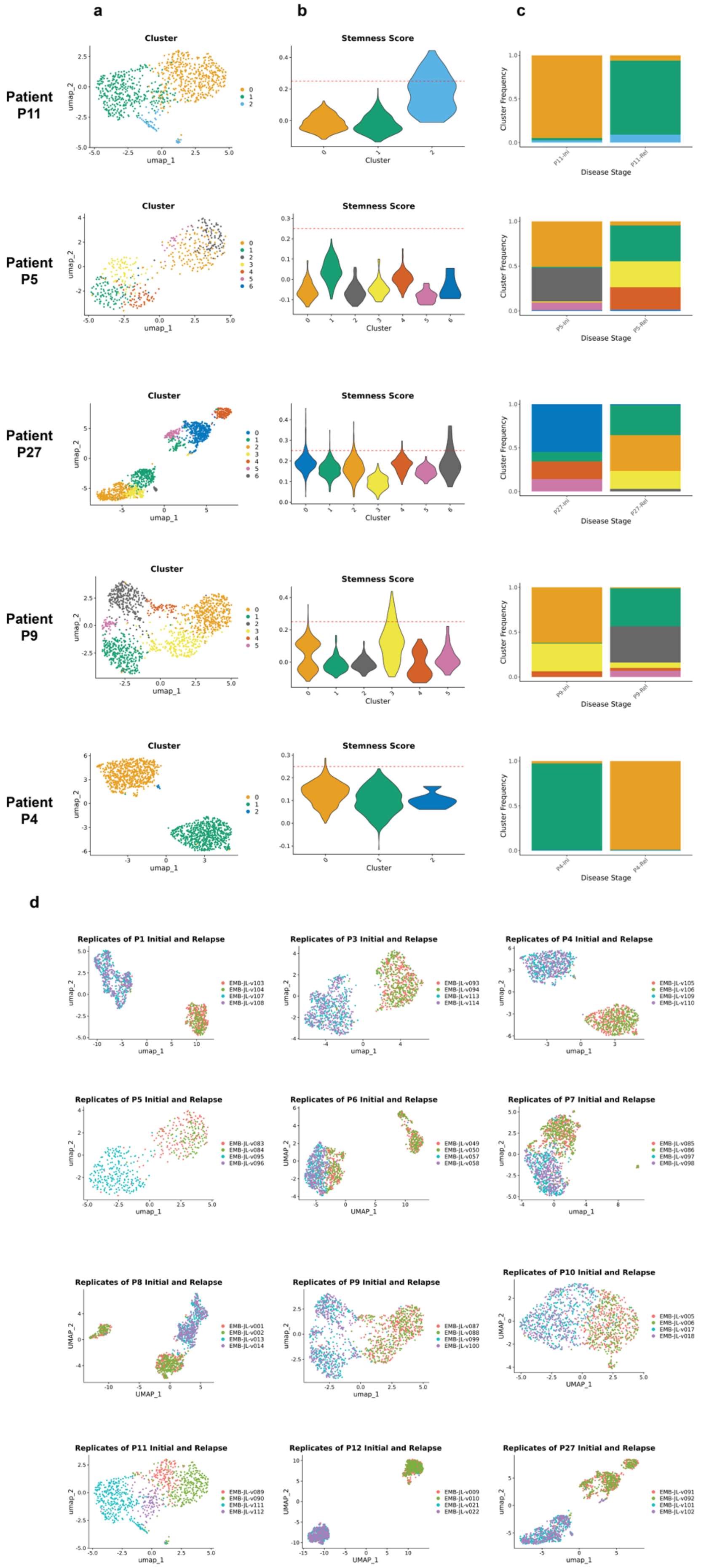
a
b
c
Patient P11
Patient P5
Patient P27
Patient P9
Patient P4
Cluster
Stemness Score
Cluster Frequency
Disease Stage
umap_1
umap_2
d
Replicates of P1 Initial and Relapse
EMB-JL-v103
EMB-JL-v104
EMB-JL-v107
EMB-JL-v108
Replicates of P3 Initial and Relapse
EMB-JL-v093
EMB-JL-v094
EMB-JL-v113
EMB-JL-v114
Replicates of P4 Initial and Relapse
EMB-JL-v105
EMB-JL-v106
EMB-JL-v109
EMB-JL-v110
Replicates of P5 Initial and Relapse
EMB-JL-v083
EMB-JL-v084
EMB-JL-v095
EMB-JL-v096
Replicates of P6 Initial and Relapse
EMB-JL-v049
EMB-JL-v050
EMB-JL-v057
EMB-JL-v058
Replicates of P7 Initial and Relapse
EMB-JL-v085
EMB-JL-v086
EMB-JL-v097
EMB-JL-v098
Replicates of P8 Initial and Relapse
EMB-JL-v001
EMB-JL-v002
EMB-JL-v013
EMB-JL-v014
Replicates of P9 Initial and Relapse
EMB-JL-v087
EMB-JL-v088
EMB-JL-v099
EMB-JL-v100
Replicates of P10 Initial and Relapse
EMB-JL-v005
EMB-JL-v006
EMB-JL-v017
EMB-JL-v018
Replicates of P11 Initial and Relapse
EMB-JL-v089
EMB-JL-v090
EMB-JL-v111
EMB-JL-v112
Replicates of P12 Initial and Relapse
EMB-JL-v009
EMB-JL-v010
EMB-JL-v021
EMB-JL-v022
Replicates of P27 Initial and Relapse
EMB-JL-v091
EMB-JL-v092
EMB-JL-v101
EMB-JL-v102

Supplementary Figure 5: **Clonal composition in PDXs of relapsing patients with <5% stem-like cells and technical replicates in PDXs of all relapsing patients.** a) Graph-based clustering analysis based on RNA expression performed for PDXs of individual patients visualized by UMAP. Number of cells per UMAP: P11 (n = 868 cells from two samples), P5 (n = 484 cells from two samples), P27 (n = 1401 cells from two samples), P9 (n = 1349 cells from two samples), P4 (n = 1298 cells from two samples). b) Stemness score of clusters in individual patients displayed by violin plots. Red dashed line reflects the threshold used for the definition of stem-like cells (0.25) (see Fig. 3e). c) Stacked barplots display the frequency of clusters at the time of initial diagnosis and relapse in PDXs of individual patients. d) UMAP plots display technical replicates for PDXs of each relapsing patient (except P2, see Supplementary Fig 1c). Number of cells per UMAP: P1 (1,042 from two samples), P3 (n = 1,355 from two samples), P4 (n = 1298 cells from two samples), P5 (n = 484 cells from two samples), P6 (n = 1,204 from two samples), P7 (n= 1,369 from two samples), P8 (n = 1,276 from two samples), P9 (n = 1349 cells from two samples), P10 (n = 1,330 from two samples), P11 (n = 868 cells from two samples), P12 (n = 1,298 from two samples), P27 (n = 1401 cells from two samples). Source data are provided as a Source Data file.

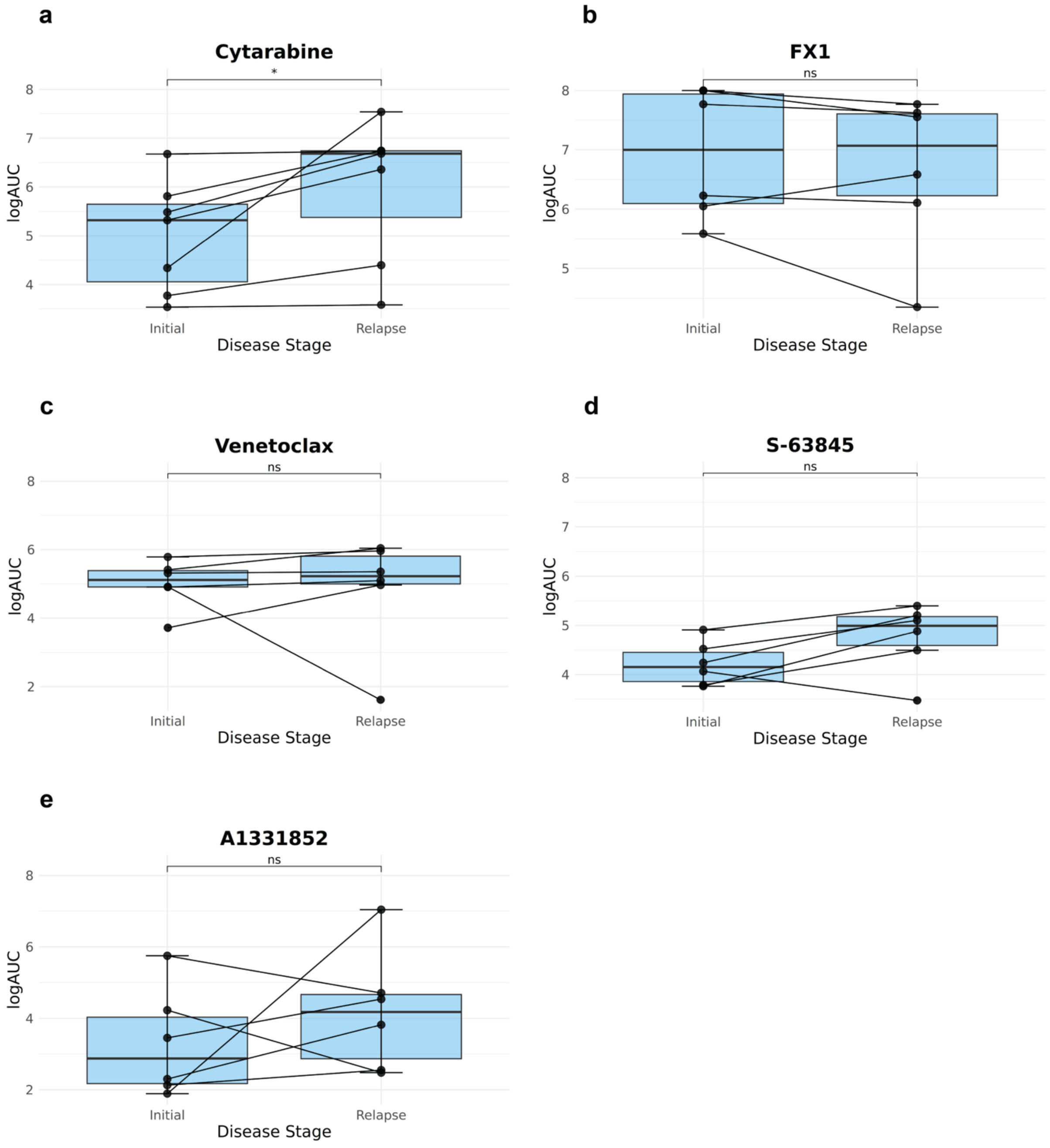

Supplementary Figure 6: ***In-vitro* drug response profiles of T-ALL PDXs at initial and relapse stage**. Boxplot displays changes in the cell viability (y-axis: logAUC) between initial disease and relapse (n =12 samples, 6 patients) following 3 days of treatment. Cells have been treated with a) Cytarabine, b) BCL-6 inhibitor FX1, c) BCL-2 inhibitor Venetoclax, d) MCL1 inhibitor S-63845, e) BCL-xL inhibitor A1331852. Boxplots show median (center line), interquartile range (box limits), whiskers extend to 1.5× IQR, and points represent outliers.

Significance was evaluated using a two-sided paired t-test. * = $p < 0.05$. Source data are provided as a Source Data file.

Supplementary References

1. Schmitz, M. *et al.* Xenografts of highly resistant leukemia recapitulate the clonal composition of the leukemogenic compartment. *Blood* **118**, 1854–1864 (2011).
2. Mihara, K. *et al.* Development and functional characterization of human bone marrow mesenchymal cells immortalized by enforced expression of telomerase. *Br. J. Haematol.* **120**, 846–849 (2003).
3. Salmen, F. *et al.* High-throughput total RNA sequencing in single cells using VASA-seq. *Nat. Biotechnol.* **40**, 1780–1793 (2022).
4. Butler, A., Hoffman, P., Smibert, P., Papalexi, E. & Satija, R. Integrating single-cell transcriptomic data across different conditions, technologies, and species. *Nat. Biotechnol.* **36**, 411–420 (2018).
5. Mathioudaki, A. *et al.* The remission status of AML patients after allo-HCT is associated with a distinct single-cell bone marrow T-cell signature. *Blood* **143**, 1269–1281 (2024).
6. Aibar, S. *et al.* SCENIC: single-cell regulatory network inference and clustering. *Nat. Methods* **14**, 1083–1086 (2017).
7. Van De Sande, B. *et al.* A scalable SCENIC workflow for single-cell gene regulatory network analysis. *Nat. Protoc.* **15**, 2247–2276 (2020).
8. Pölönen, P. *et al.* The genomic basis of childhood T-lineage acute lymphoblastic leukaemia. *Nature* **632**, 1082–1091 (2024).
9. Fang, Z., Liu, X. & Peltz, G. GSEApy: a comprehensive package for performing gene set enrichment analysis in Python. *Bioinformatics* **39**, btac757 (2023).
10. Park, J.-E. *et al.* A cell atlas of human thymic development defines T cell repertoire formation. *Science* **367**, eaay3224 (2020).

11. Hao, Y. *et al.* Integrated analysis of multimodal single-cell data. *Cell* **184**, 3573-3587.e29 (2021).
12. Pertea, M., Kim, D., Pertea, G. M., Leek, J. T. & Salzberg, S. L. Transcript-level expression analysis of RNA-seq experiments with HISAT, StringTie and Ballgown. *Nat. Protoc.* **11**, 1650–1667 (2016).
13. Parada, G. E. *et al.* MicroExonator enables systematic discovery and quantification of microexons across mouse embryonic development. *Genome Biol.* **22**, 43 (2021).
14. Garrido-Martín, D., Palumbo, E., Guigó, R. & Breschi, A. ggsashimi: Sashimi plot revised for browser- and annotation-independent splicing visualization. *PLOS Comput. Biol.* **14**, e1006360 (2018).
15. ElTanbouly, M. A. *et al.* VISTA is a checkpoint regulator for naïve T cell quiescence and peripheral tolerance. *Science* **367**, eaay0524 (2020).
16. Paugh, S. W. *et al.* NALP3 inflammasome upregulation and CASP1 cleavage of the glucocorticoid receptor cause glucocorticoid resistance in leukemia cells. *Nat. Genet.* **47**, 607–614 (2015).
17. Bortolozzi, R. *et al.* AKR1C enzymes sustain therapy resistance in paediatric T-ALL. *Br. J. Cancer* **118**, 985–994 (2018).
