# Supplementary material for: Role of stem-like cells in chemotherapy resistance and relapse in pediatric T-cell acute lymphoblastic leukemia
Source: Nat Commun. 2025 Jun 27;16:5413. doi: 10.1038/s41467-025-61222-1 (PMC12205070; doi:10.1038/s41467-025-61222-1)
Supplement: Supplementary file 2 — Description of Additional Supplementary Files [file 41467_2025_61222_MOESM2_ESM.pdf]

Description of Additional Supplementary Files:

Supplementary Data 1: Patients' clinical characteristics

Supplementary Data 2: Regulon activity (pySCENIC) of P2 clusters

Supplementary Data 3: Differential expression markers of TAL1 stem-like cells vs other leukemic blasts

Supplementary Data 4: Regulon activity (pySCENIC) of TAL1 stem-like cells vs other leukemic blasts

Supplementary Data 5: Differential analysis of regulon activity (pySCENIC) between individual patients

Supplementary Data 6: Metadata and list of events of AS analysis

Supplementary Data 7: Differential expression markers of PDXs from individual patients

Supplementary Data 8: *In-vitro* drug response parameters
