# Supplementary material for: Role of stem-like cells in chemotherapy resistance and relapse in pediatric T-cell acute lymphoblastic leukemia
Source: Nat Commun. 2025 Jun 27;16:5413. doi: 10.1038/s41467-025-61222-1 (PMC12205070; doi:10.1038/s41467-025-61222-1)
Supplement: Supplementary file 11 — Reporting Summary [file 41467_2025_61222_MOESM11_ESM.pdf]

nature portfolio

Corresponding author(s): Andreas Kulozik & Jan Korbel

Last updated by author(s): 5/29/2025

Reporting Summary

Nature Portfolio wishes to improve the reproducibility of the work that we publish. This form provides structure for consistency and transparency in reporting. For further information on Nature Portfolio policies, see our Editorial Policies and the Editorial Policy Checklist.

Statistics

For all statistical analyses, confirm that the following items are present in the figure legend, table legend, main text, or Methods section.

n/a	Confirmed	
☐	☒	The exact sample size (*n*) for each experimental group/condition, given as a discrete number and unit of measurement
☐	☒	A statement on whether measurements were taken from distinct samples or whether the same sample was measured repeatedly
☐	☒	The statistical test(s) used AND whether they are one- or two-sided<br>*Only common tests should be described solely by name; describe more complex techniques in the Methods section.*
☒	☐	A description of all covariates tested
☐	☒	A description of any assumptions or corrections, such as tests of normality and adjustment for multiple comparisons
☐	☒	A full description of the statistical parameters including central tendency (e.g. means) or other basic estimates (e.g. regression coefficient) AND variation (e.g. standard deviation) or associated estimates of uncertainty (e.g. confidence intervals)
☐	☒	For null hypothesis testing, the test statistic (e.g. *F*, *t*, *r*) with confidence intervals, effect sizes, degrees of freedom and *P* value noted<br>*Give P values as exact values whenever suitable.*
☒	☐	For Bayesian analysis, information on the choice of priors and Markov chain Monte Carlo settings
☒	☐	For hierarchical and complex designs, identification of the appropriate level for tests and full reporting of outcomes
☐	☒	Estimates of effect sizes (e.g. Cohen's *d*, Pearson's *r*), indicating how they were calculated

Our web collection on statistics for biologists contains articles on many of the points above.

Software and code

Policy information about availability of computer code

Data collection: Our study have used sequencing data output by illumina sequencers and no special software was used for collecting it.

Data analysis: Code Availability
The computational workflow related to the alternative splicing analysis can be found here: https://github.com/Zaffe24/AS_VASAseq_sc_pipeline (https://doi.org/10.5281/zenodo.1551214353). All other scripts related to the analysis of this study can be found here: https://github.com/Zaffe24/Costea-et-al.-2024 (https://doi.org/10.5281/zenodo.15514411).
Software includes: BD FACSDiva, Seurat (version 5.1.0), dittoSeq (version 1.10.0), ggplot2(version 3.5.1.9000), stringr(1.5.1), dplyr (version 1.1.4), scProportionTest (version 0.0.0.9000), Trim Galore (version 0.6.7), Cutadapt (version 3.5), bwa (version 0.7.17), Samtools (version 1.16.1), BBMap (version 38.98), Snakemake (version 7.32.4), hisat2 (version 2.1.0), StringTie2 (version 2.2.1), gffcompare (version 0.12.6), MicroExonator (version 1.0.0), Whippet (version 1.6.1), gseapy (version 1.1.1), matplotlib (version 3.8.2), scanpy (version 1.9.8), ggsashimi (version 1.1.5), pyscenic (version 0.12.1), arboreto (version 0.1.6).

For manuscripts utilizing custom algorithms or software that are central to the research but not yet described in published literature, software must be made available to editors and reviewers. We strongly encourage code deposition in a community repository (e.g. GitHub). See the Nature Portfolio guidelines for submitting code & software for further information.

Data

Policy information about availability of data

All manuscripts must include a data availability statement. This statement should provide the following information, where applicable:

- Accession codes, unique identifiers, or web links for publicly available datasets
- A description of any restrictions on data availability
- For clinical datasets or third party data, please ensure that the statement adheres to our policy

The publicly available RNA-seq data used in this study are available from the database of Genotypes and Phenotypes (dbGaP) under accession number phs002276.v2.p1. The scRNA-seq data generated in this study have been deposited in the European Genome-phenome Archive database under accession code EGAS50000000582. Due to the sensitive nature of human genomic data and to protect participant privacy, the data is access-controlled. Data access is governed by the Data Access Committee (DAC), which ensures compliance with ethical, legal, and institutional guidelines. Researchers seeking access must submit a data access request via the EGA platform, outlining the intended use of the data. Access will be granted to researchers affiliated with academic or research institutions and requests will typically be reviewed and responded to within 10 working days. Once approved, data will be available to the requestor until project completion. The remaining data are available within the Article, Supplementary Information or Source Data file.

Research involving human participants, their data, or biological material

Policy information about studies with human participants or human data. See also policy information about sex, gender (identity/presentation), and sexual orientation and race, ethnicity and racism.

Reporting on sex and gender	In our study patient selection was not based on sex. Sex-based analyses are not performed in this study. Demographic data including sex and age can be found in supplementary table 1.
Reporting on race, ethnicity, or other socially relevant groupings	-
Population characteristics	Relapsing patient samples have been collected at the time of initial diagnosis and relapse. Non-relapsing patients samples have been collected at initial diagnosis.
Recruitment	Samples have been taken from patients that participated in these clinical trials: ALL-BFM 2000 (Conter et al, 2010, Blood PMID: 20154213) , ALL-BFM 2009 (Campbell et al 2013, ASCO Journal of Clinical Oncology, PMID: 37141547), CoALL03 (Schramm et al 2019, Blood Advances, PMID: 31765480), CoALL09 (Escherich et al 2022, Haematologica, PMID: 34348455).
Ethics oversight	Clinical trials from which samples were used in this analysis had previously received approval from the relevant institutional review boards or ethics committees. Written informed consent had been obtained from all the patients or legal guardians, and the experiments conformed to the principles set out in the WMA Declaration of Helsinki and the Department of Health and Human Services Belmont Report.

Note that full information on the approval of the study protocol must also be provided in the manuscript.

Field-specific reporting

Please select the one below that is the best fit for your research. If you are not sure, read the appropriate sections before making your selection.

[x] Life sciences [ ] Behavioural & social sciences [ ] Ecological, evolutionary & environmental sciences

For a reference copy of the document with all sections, see nature.com/documents/nr-reporting-summary-flat.pdf

Life sciences study design

All studies must disclose on these points even when the disclosure is negative.

Sample size	This study includes patient derived xenograft samples from 5 non-relapsing patients at initial diagnosis and 13 relapsing patients at initial diagnosis and relapse.
Data exclusions	No data was excluded from the analysis.
Replication	Technical replicates for VASA-seq analysis have been generated for all patients.
Randomization	-
Blinding	-

Reporting for specific materials, systems and methods

We require information from authors about some types of materials, experimental systems and methods used in many studies. Here, indicate whether each material, system or method listed is relevant to your study. If you are not sure if a list item applies to your research, read the appropriate section before selecting a response.

Materials & experimental systems

n/a	Involved in the study
☐	☒ Antibodies
☐	☐ Eukaryotic cell lines
☐	☐ Palaeontology and archaeology
☐	☒ Animals and other organisms
☐	☒ Clinical data
☒	☐ Dual use research of concern
☒	☐ Plants

Methods

n/a	Involved in the study
☒	☐ ChIP-seq
☐	☒ Flow cytometry
☒	☐ MRI-based neuroimaging

Antibodies

Antibodies used	FACS: anti-mouse CD45 (eFluor 450, eBiosciences, 1:100), anti-human CD7 (PE, eBiosciences, 1:25), anti-human CD45 (Alexa Fluor 647, BioLegend, 1:25), anti-mouse CD45-PE (mCD45) (clone 30-F11; BioLegend; 1:20)
Validation	All antibodies were validated for the specific application by the manufacturer and validation data is available on the manufacturer's website.

Eukaryotic cell lines

Policy information about cell lines and Sex and Gender in Research

Cell line source(s)	-
Authentication	-
Mycoplasma contamination	-
Commonly misidentified lines (See ICLAC register)	-

Palaeontology and Archaeology

Specimen provenance	-
Specimen deposition	-
Dating methods	-

☐ Tick this box to confirm that the raw and calibrated dates are available in the paper or in Supplementary Information.

Ethics oversight	-

Note that full information on the approval of the study protocol must also be provided in the manuscript.

Animals and other research organisms

Policy information about studies involving animals; ARRIVE guidelines recommended for reporting animal research, and Sex and Gender in Research

Laboratory animals	NSG (NOD.Cg-Prkdscidll2rgtm1Wjl/SzJ) mice
Wild animals	-
Reporting on sex	female and male
Field-collected samples	-

Ethics oversight	In-vivo experiments were approved by the veterinary office of the Canton of Zurich, in compliance with ethical regulations for animal research.

Note that full information on the approval of the study protocol must also be provided in the manuscript.

Clinical data

Policy information about clinical studies

All manuscripts should comply with the ICMJEguidelines for publication of clinical research and a completedCONSORT checklist must be included with all submissions.

Clinical trial registration	ALL-BFM 2000: https://www.gpoh.de/studienportal/abgeschlossene_studien_register/all_bfm_2000/index_ger.html, ALL-BFM 2009: https://www.gpoh.de/studienportal/abgeschlossene_studien_register/aieop_bfm_all_2009/index_ger.html, CoALL03: https://www.gpoh.de/studienportal/abgeschlossene_studien_register/coall_07_03/index_ger.html, CoALL09: https://www.gpoh.de/studienportal/abgeschlossene_studien_register/coall_08_09/index_ger.html
Study protocol	The patients were treated according to the respective protocols (please find study protocols under the website mentioned in the above clinical trial registration section)
Data collection	ALL BFM 2000: children and adolescents ≤18 years with ALL. 2000 patients recruited (start 01.12.2000, end: 30.06.2009).<br>ALL BFM 2009: Children and adolescents ≥1 year and ≤ 18 years with ALL. 5000 patients recruited (end 31.23.2016).<br>CoALL03: Children and adolescents ≤ 18 years with precusor B-ALL or T-ALL. 110 patients recruited each year (start 01.09.2003-31.08.2015).<br>CoALL09: Children and adolescents ≤ 18 years with precursor B-ALL or T-ALL. 110 patients recruited each year (start 01.10.2010, end: 31.12.2019).<br>Please find further details under the websiste mentioned in the above clinical trial registration section.
Outcomes	Primary and secondary outcomes can be found on the websites mentioned in the above clinical trial registration section as well as in the publications mentioned in the recruitment section above.

Plants

Seed stocks	-
Novel plant genotypes	-
Authentication	-

Flow Cytometry

Plots

Confirm that:

- ☐ The axis labels state the marker and fluorochrome used (e.g. CD4-FITC).
- ☐ The axis scales are clearly visible. Include numbers along axes only for bottom left plot of group (a 'group' is an analysis of identical markers).
- ☐ All plots are contour plots with outliers or pseudocolor plots.
- ☐ A numerical value for number of cells or percentage (with statistics) is provided.

Methodology

Sample preparation	Cryopreserved cells were thawed at 37°C and resuspended in 10 ml RPMI medium + 20 % FBS. Cells were centrifuged for 5 min at 300g and resuspended in ice-cold PBS + 2% FBS + 5mM EDTA. Cells were stained on ice and in the dark for 30 min with anti-murine-CD45-PE (mCD45)(clone 30-F11; BioLegend; 1:20). 1:100 4,6-diamidino-2-phenylindole (DAPI) was added immediately before FACS sorting. mCD45-DAPI- cells were sorted using a BD FACSAria fusion cell sorter.
Instrument	BD FACSAria fusion cell sorter
Software	FlowJo, BD FACSDiva
Cell population abundance	The cell population abundance depends on the samples. Specific cell populations were identified through surface marker staining and flow cytometric identification.

Gating strategy

FSC and SSC were applied to set the gate for single cell populations. DAPI staining was applied to exclude dead cells and mCD45-PE antibody was used to exclude potential mouse cells.

☐ Tick this box to confirm that a figure exemplifying the gating strategy is provided in the Supplementary Information.
